# Epithelial Interleukin‐1 Receptor‐Like‐1 Activation Is Contingent on Interleukin‐33 Isoforms and Asthma‐Related Receptor Variation

**DOI:** 10.1111/cea.14562

**Published:** 2024-09-20

**Authors:** Michael A. Portelli, Maria E. Ketelaar, Stewart Bates, Eszter Csomor, Dominick Shaw, Jonas Emsley, Christopher Brightling, Ian Hall, Karen Affleck, Matthew Edwards, Martijn C. Nawijn, Gerard H. Koppelman, Antoon J. Van Oosterhout, Ian Sayers

**Affiliations:** ^1^ Centre for Respiratory Research, National Institute for Health Research Nottingham Biomedical Research Centre, School of Medicine, Biodiscovery Institute University of Nottingham Nottingham UK; ^2^ Groningen Research Institute for Asthma and COPD, Department of Pediatric Pulmonology and Pediatric Allergology, Beatrix Children's Hospital University of Groningen, University Medical Center Groningen Groningen The Netherlands; ^3^ Groningen Research Institute for Asthma and COPD, Department of Pathology and Medical Biology University of Groningen, University Medical Center Groningen Groningen The Netherlands; ^4^ UK Respiratory Therapeutic Unit GlaxoSmithKline pPlc, 1929 Brentford UK; ^5^ School of Pharmacy, Biodiscovery Institute University of Nottingham Nottingham UK; ^6^ Respiratory Sciences Glenfield Hospital, University of Leicester Leicester UK; ^7^ Allergic Inflammation Discovery Performance Unit GlaxoSmithKline Stevenage UK

**Keywords:** IL1RL1, IL33, signalling, variation

## Abstract

**Introduction:**

The interleukin‐33/interleukin‐1 receptor‐like‐1 (IL‐33/IL1RL1) signalling pathway is implicated in asthma pathogenesis, with *IL1RL1* nonsynonymous genetic polymorphisms associated with disease risk. We aimed to determine these variants' effect on IL1RL1 signalling induced by different IL33 isoforms thought to be elevated in the asthmatic airway.

**Method:**

In a project funded by GSK plc, which has developed an IL‐33 receptor inhibitor for asthma treatment, human embryonic kidney 293 (HEK293) cells expressing secreted embryonic alkaline phosphatase (SEAP) driven by a nuclear factor kappa‐beta (NF‐κB) promoter, were transiently transfected with *IL1RL1*, containing one of four extracellular and Toll/interleukin 1 receptor (TIR) domain haplotypes. Cells were stimulated with seven different splice and proteolytic‐generated IL‐33 isoforms (0.001–50 ng/mL) for 24 h. Supernatant SEAP activity and interleukin‐8 (IL‐8) levels were determined. Primary human bronchial epithelial cells (HBECs) representing different genotype carriers were stimulated with IL‐33_112–270_ (50 ng/mL) and induced IL‐8 mRNA expression measured.

**Results:**

HEK293 cells carrying both asthma extracellular and TIR domain *IL1RL1* risk haplotypes presented maximal IL33‐driven signalling, with minimal signalling after IL‐33 activation in other protective haplotypes. All IL‐33 isoforms activated IL1RL1 but with differing magnitudes. Proteolytically cleaved IL33_95–270_ and IL33_106–270_ had the greatest effect and the IL33_113–270_, and Exon 3,4 deletion isoform exhibited the lowest. The effect of extracellular and TIR domain genetic variants on receptor signalling was replicated in primary HBECs. Maximal IL1RL1 signalling was observed in cells carrying both extracellular and TIR signalling domain risk haplotypes.

**Conclusions:**

Overall, our study suggests asthma patients carrying the extracellular and TIR domain risk haplotype and have a lung microenvironment that promotes elevated levels of cleaved IL33, particularly where IL33_95–270_ and IL33_106–270_ may be more amenable to IL33/IL1RL1 targeting.

## Graphical abstract

IL‐33 isoforms generated through allergen‐driven proteolysis, signal through NF‐κB at varying intensities. The magnitude of IL‐33 signalling is determined by asthma‐associated structural variation in IL33 receptor. Variation in the IL‐33 receptor extracellular domain alters accessibility of the IL‐33 ligand binding site.

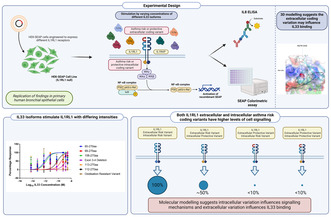

**Abbreviations**
AP‐1: activator protein 1
CG: cathepsin‐G
GINA: Global Initiative for Asthma
HBEC: Human Brochial Epithelial Cells
IL1RL1: interleukin 1 receptor‐like 1
IL8: interleukin 8
IL33: interleukin 33
IRAK: IL‐1R‐associated kinase
NE: neutrophil elastase
NF‐κB: nuclear factor‐κB
PDB: protein DataBank
SEAP: secreted embryonic alkaline phosphatase
TIR: Toll–IL‐1 receptor
Traf: TNFR‐associated factor

**Summary**

- IL‐33 isoforms generated through allergen‐driven proteolysis, signal through NF‐κB at varying intensities.
- The magnitude of IL‐33 signalling is determined by asthma‐associated structural variation in IL33 receptor.
- Variation in IL‐33 receptor extracellular domain potentially alters accessibility of the IL‐33 ligand binding site.

## 1 Introduction

Interleukin 33 (IL33) is an IL‐1 family alarmin found in epithelial cell nuclei and released into the extracellular compartment upon cellular damage, where it interacts with interleukin‐1 receptor‐like 1 (*IL1RL1*; ST2), initiating signalling through a Toll IL‐1 receptor (TIR) domain via a heterodimeric receptor complex with IL‐1 receptor accessory protein (IL1RAP). Subsequent activation of MyD88 and IL‐1R‐associated kinase (IRAK) leads to activation of activator protein 1 (AP‐1) and liberation of active nuclear factor‐κB (NF‐κB) [1]. This drives Type 2 inflammation, with implications for related diseases, for example, asthma, through basophil, Innate Type 2 lymphoid cell (ILC2) and mast cell activation.

The IL33/IL1RL1 axis represents a therapeutic opportunity for asthma and allergic diseases and is of interest for several pharmaceutical companies developing selective inhibitors/blocking antibodies. These include GSK's anti‐IL1RL1 GSK3772847 (CNTO7160), Regeneron's anti‐IL33 REGN3500 (SAR440340), Amgen's anti‐IL1RL1 AMG282 (RG6149/ MSTT1041A) and AnaptysBio's anti‐IL33 Etokimab (ANB020) [2, 3].

Together with the *IL33* locus, one of the most highly reproduced association signals in genome‐wide association studies of asthma, spans the *IL1RL1 gene* [4]. These include candidate causal nonsynonymous variation, including five Exon 11 SNPs in complete linkage disequilibrium, coding for a four amino acid haplotype in the TIR signalling domain (Ala433Thr/Gln501Arg/Thr549Ile/Leu551Ser) [5, 6, 7] and an Exon 3 variant (rs1041973) which modifies an amino acid (Ala78Glu) within the first immunoglobulin‐like domain of the receptor's extracellular domain [6, 8, 9]. We and others have reported that the TIR domain asthma risk haplotype increased IL33_112–270_‐driven signalling [10, 11, 12], however the functional effect of the Ala78Glu variant in isolation or in combination with the TIR domain variation has not been studied to date.

Full‐length IL‐33_1–270_ undergoes a process of proteolytic cleavage via proteases, including cathepsin‐G (CG) and neutrophil elastase (NE). These neutrophil serine proteases, released via asthma‐relevant allergens, for example, fungi, house dust mites and viruses, generate several IL33 isoforms detected in the airways and which have biological activity [13, 14, 15]. Importantly, these IL33 isoforms (IL‐33_95–270_, IL‐33_107–270_, and IL‐33_109–270_) present with increased abundance in the airways due to increased protease expression of, for example, mast cell‐derived chymase and tryptase. These forms show increased potency (30‐fold) than IL33_1–270_ in activating IL1RL1 on, for example, ILC2 cells leading to IL4, IL5, IL13 production [16]. In addition to these proteolytically cleaved IL33 isoforms, several IL33 splice variants that translate into truncated proteins also exist. Of these, an Exon 3,4 deletion variant (IL33_Δ3,4_) has been associated with airway Type 2 inflammation of relevance to asthma and has been shown to be expressed in the airway epithelium with biological activity [17]. To our knowledge, the functional relevance of these different IL33 isoforms in combination with naturally occurring *IL1RL1* nonsynonymous variation remains undefined.

We hypothesised that different isoforms of IL33, that may be more prevalent in the airways of patients with asthma, may differentially activate IL1RL1 and that asthma‐associated *IL1RL1* nonsynonymous variation may further modify the magnitude of this signalling response. These data have broad implications for identifying potentially ‘IL33‐driven’ asthma subtypes and hence patients more amenable to targeted anti‐IL33/IL1RL1 treatment.

## 2 Methods

See Appendix S1 for additional details.

### 2.1 Cell Lines

Human NF‐κB secreted alkaline phosphatase reporter SEAP—(SEAPorter) stable reporter cells (NovusBio NBP2‐26260) were cultured in DMEM containing 4.5 g/L glucose and 4 mM L‐glutamine (Sigma D5796) supplemented with 10% FBS, 1 mM sodium pyruvate, 100 units/mL Penicillin, 100 μg/mL Streptomycin and 500 μg/mL G418. Cells were transfected with the *IL1RL1* plasmids in a 96‐well plate format using the TransIT‐LT1 lipid transfection reagent (Mirus Bio, MIR2300) as per manufacturer's instructions. An empty vector was used as a transfection control and quantity adjustments were made to correct for differences in plasmid size. Receptor expression levels were determined using Taqman qPCR as previously described (Figure S1). Following a 24 h period, cells were stimulated with a range of concentrations (0.0001, 0.001, 0.01, 0.1, 1, 10, 25 and 50 ng/mL) of the six generated human recombinant IL‐33 proteins and a commercially available isoform spanning amino acids 112–270 (ABNOVA, P3638). Appropriate media and vehicle (PBS) controls, as well as a positive 10 ng/μL TNFα control (activates NF‐κB), were also included. Cell supernatants were collected following a 24‐h incubation.

### 2.2 Generating IL33 Isoforms

Recombinant IL33 proteins were generated using vector‐driven expression in *E. coli* and affinity column purified. These were the 95–270aa, 99–270aa, 109–270aa and 113–270aa isoforms, an Exon 3–4 deletion variant and a 113–270aa oxidation‐resistant isoform. Cysteine oxidation has been reported to limit IL33 activity through the formation of disulphide bridges within the protein structure [18]. These changes to the conformation of IL33 inhibits IL1RL1 binding, resulting in attenuated signalling. We therefore generated an oxidation‐resistant IL33_113–270_ (IL33_OxR_) isoform for inclusion in all experiments.

### 2.3 Quantification of IL8 mRNA Expression

HBEC complimentary DNA (cDNA) was synthesised from 1 μg RNA using Superscript II (Invitrogen; Paisley, UK) and random hexamer primers according to the manufacturer's instructions. *IL8* mRNA levels determined using a TaqMan quantitative PCR assay (Hs00174103_m1, Applied Biosystems) using TaqMan gene expression master mix (Applied Biosystems) and *HPRT1* endogenous control (Hs01003267_m1, Applied Biosystems) on a Stratagene MxPro3005 machine using 40 cycles of 95°C for 15 s and 60°C for 60 s. Data were normalised using the housekeeper (HPRT1) and the 2^−Δ*Ct*^ method.

### 2.4 Genotyping Bronchial Epithelial Cells

DNA was extracted using the Qiagen QIAamp DNA Mini and Blood Mini Kit according to the manufacturer's instructions. SNP Genotyping was then carried out using TaqMan Predesigned assays (Applied Biosystems, 4351379).

### 2.5 Analysing NF‐κB Activity

NF‐κB‐activated SEAP release was measured 24 h following IL‐33 stimulation, using a commercial SEAP Reporter Assay Kit (Invivogen, rep‐sap). Ten microliters of cellular supernatant was used and activity measured as per manufacturer's instructions.

### 2.6 IL8 ELISA

IL8 levels were determined using a Duoset ELISA (R&D Systems; Abingdon, UK). Samples were diluted 1:2 in reagent diluent. Assays were read at 450 nm (background subtraction 570 nm), using a Flexstation 3 microplate reader (Molecular Devices; Wokingham, UK).

### 2.7 Modelling the Structural Impact of Nonsynonymous Amino Acid Changes

We examined the predicted structural effects of the Ala78Glu substitution. The coordinates for the *IL1RL1* complex structure were downloaded from the Protein DataBank (PDB) (Code: 4KC3). The Ala78Glu substitution in *IL1RL1* was generated using the mutate function of COOT [19].

## 3 Results

### 3.1 IL33 Isoforms Are Able to Activate the IL1RL1 Signalling Pathway

**FIGURE 1:**
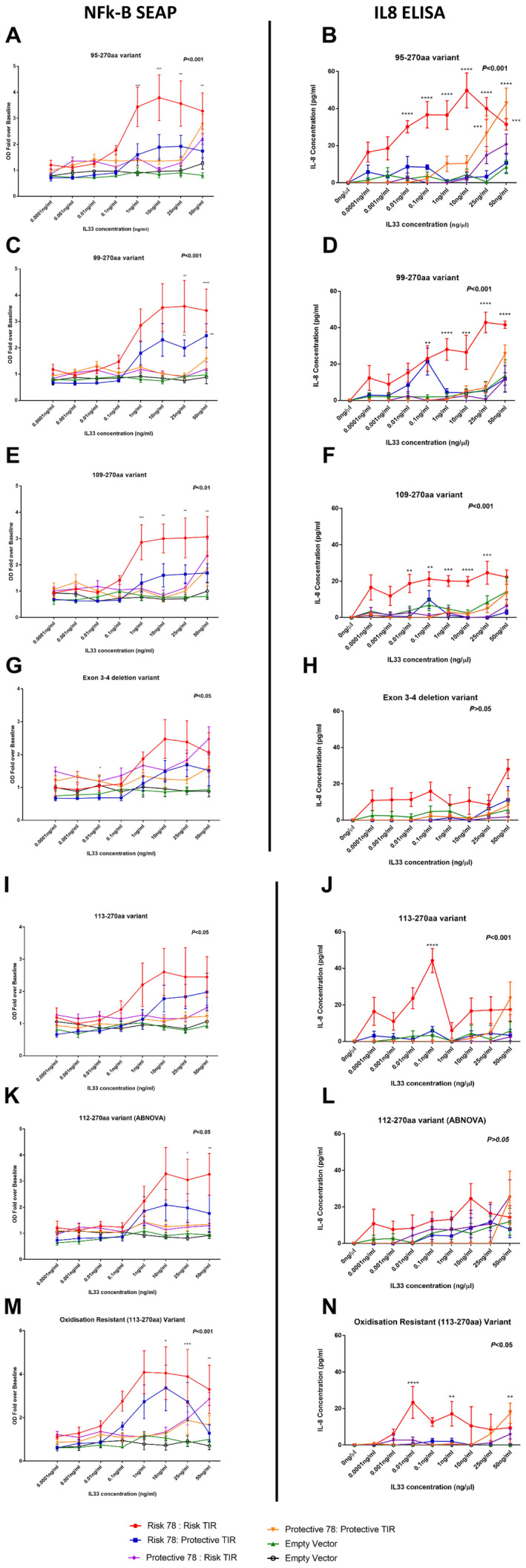
IL33 dose–response curves identify different signalling patterns dependent on IL33 isoform studied and *IL1RL1* genotype. Stimulation of recombinant HEK293 cells stably transfected to express SEAP through and *NF‐κB* promoter with different isoforms of IL33 following transient transfection of *IL1RL1* identifies that both risk alleles/haplotypes are required for IL33 driven signalling. While all IL33 isoforms show some degree of signalling, the strongest responses are observed with IL33_95–270_ (Panels A,B), IL33_99–270_ (Panels C,D) and IL33_106–270_ (Panels E,F), presenting with both the highest readings and low dose‐driven responses over baseline. All SEAP responses (Panels A, C, E, G, I, K) were confirmed by measuring IL8 levels (Panels B, D, F, H, J, L) except for responses driven by IL33_OxR_ (Panels M,N). Data represent means ± SEM (*n* = 5 independent experiments). Comparisons were analysed using a two‐way ANOVA with Sidak correction for multiple testing. Presented *p* values represent the ANOVA statistical output. *p < 0.05, **p < 0.01, ***p < 0.001, ****p < 0.0001.

In the HEK‐293 IL1RL1 reporter system, SEAP activity levels in cellular supernatants identified that the majority of IL33 isoforms (IL33_95–270_, IL33_99–270aa_, IL33_109–270aa_, IL33_112–270aa_ [ABNOVA] and IL33_OxR_) were able to activate the NF‐κB pathway through IL1RL1 signalling (Figure 1, Panels A,C,E,G,I,K,M, *p* < 0.05) 24‐h poststimulation. A modest increase in SEAP activity was observed for isoforms IL33_Δ3,4_ and IL33_113–270_, which did not reach statistical threshold when compared to the control condition (Figure 1, Panel G). IL1RL1 mRNA levels were not different across all variant receptor transfections and TNFα induced SEAP activity levels were identical across all cell lines, highlighting that all cells had the same level of IL1RL1 mRNA and capacity to signal via the NF‐κB pathway and this did not confound the interpretation of results (Figures S1 and S2).

When measuring IL1RL1 signalling via direct measurement of IL8 levels in the same supernatants used to measure SEAP activity (Figure 1, Panels B,D,F,H,J,L,N), we observed a similar response to that in the SEAP assay, where signalling was observed for the IL33_95–270_, IL33_99–270aa_, IL33_109–270aa_, IL33_112–270aa_ and the IL33_Δ3,4_ isoform (*p* < 0.05). For the IL33_112–270aa_ isoform and in contrast to SEAP recorded data, a modest but statistically insignificant increase in IL‐8 activity was observed.

### 3.2 Maximal IL1RL1 Signalling Requires the *IL1RL1* Risk–Risk Haplotype

When comparing signalling activity over the four different IL1RL1 protein structures, we observed that a maximal response to the IL33 isoforms occurred in cells expressing IL1RL1 with both asthma risk alleles for the extracellular‐variant rs1041973 (C) and the asthma‐associated TIR signalling domain haplotype tagged by rs10192157 (C). NF‐κB signalling was only activated at higher IL33 concentrations, generally at 25 or 50 ng/mL; however, for the IL33_95–270_ and IL33_109–270_ isoforms a response could be observed at 10 and 1 ng/mL (Figure 1, Panels A,E, *p* < 0.05).

When measuring IL8 levels (Figure 1, Panels B,D,F,H,J,L,N) we observe a similar response but with a different dose–response relationship compared with the promoter–reporter (SEAP) data, that is, the presence of both the asthma risk alleles was required for maximal IL1RL1 signalling. IL33_95–270_, IL33_99–270aa_, IL33_109–270aa_, IL33_113–270_ and IL33_OxR_ activated signalling, while no statistically significant signalling was observed for IL33 _Δ3,4_ or IL33_112–270aa_, suggesting a disconnect between promoter–reporter response using the NF‐κB reporter element and secretion of an NF‐κB induced cytokine at the protein level. IL33_113–270aa_ signalling occurred at the 0.1 ng/mL concentration. A significant response was observed for a concentration of 0.01 ng/mL (the IL33_95–270_, IL33_109–270aa_ and IL33_OxR_ isoforms) or 0.1 ng/mL (IL33_99–270aa_).

In both, the SEAP assay and the IL8 ELISA, maximal responses were observed in two of the longest IL33 variants (IL33_95–270aa_, IL33_109–270aa_) as well as in the IL33_OxR_ (Table 1). Minimal responses were observed in the IL33 _Δ3,4_ variant for both SEAP (OD: 2.47) and IL8 (28.14 pg/mL) assays (Table 1).

**TABLE 1:** Maximal and EC_50_ values for IL1RL1 signalling responses dependent of TIR signalling domain haplotype.

Isoform (molarity)	SEAP maximum (OD)	SEAP EC_50_ Risk:Risk	SEAP EC_50_ Risk:Prot	*p* Risk versus Prot EC_50_	IL8 ELISA maximum (pg/μL)	IL8 EC_50_
IL33_95–270aa_ 19,826.24 g/mol	3.79 (10 ng/mL)	6.99 pM	42.70 pM	0.081	49.74 (10 ng/mL)	0.74 pM
IL33_99–270aa_ 19,437.77 g/mol	3.58 (25 ng/mL)	29.19 pM	90.58 pM	0.213	42.85 (25 ng/mL)	28.96 nM
IL33_106–270aa_ 18,333.50 g/mol	3.07 (50 ng/mL)	14.73 pM	71.19 pM	0.061	24.64 (25 ng/mL)	1.17 pM
IL33_Δ3,4_ 16,249.69 g/mol	2.47 (10 ng/mL)	22.08 pM	46.58 pM	0.391	28.15 (50 ng/mL)	1.50 nM
IL33_113–270aa_ 17,907.11 g/mol	2.60 (10 ng/mL)	13.48 pM	188.5 pM	0.110	37.81 (0.1 ng/mL)	1.58 nM
IL33_112–270aa_ 17,994.19 g/mol	3.28 (50 ng/mL)	46.16 pM	200.1 pM	0.235	24.58 (10 ng/mL)	69.80 pM
IL33_OxR_ 17,907.11 g/mol	4.09 (10 ng/mL)	3.95 pM	4.92 pM	0.851	23.34 (0.01 ng/mL)	24.8 μM

*Note:* The maximal response is generated by the IL33_95–270_ mature isoform, with a maximum SEAP OD of 3.79 and IL8 concentration of 49.74 pg/mL occurring at a concentration of 10 ng/mL. EC_50_ values (SEAP: 6.99 pM, IL8: 0.74 pM) confirm that this IL33 mature isoform as the most potent activator of IL1RL1. Although not achieving statistical significance he second highest maximal SEAP and IL8 response was IL33_106–270_ with EC_50_ values of 14.73 pM (SEAP) and 1.17 pM (IL8). Although presenting with strong responses in the SEAP assay the IL33_OxR_ isoform presents with no discernible signal when considering IL8 as a measurement of IL8 signalling, suggesting that this isoform of IL33 has limited experimental suitability. Comparisons between SEAP EC_50_ values across genotypes identify no statistically significant difference in values. Comparisons were analysed using a Mann–Whitney test. No data are presented for variants carrying the 78 variant protective allele (A) as insufficient responses to calculate an EC_50_ were observed.

**FIGURE 2:**
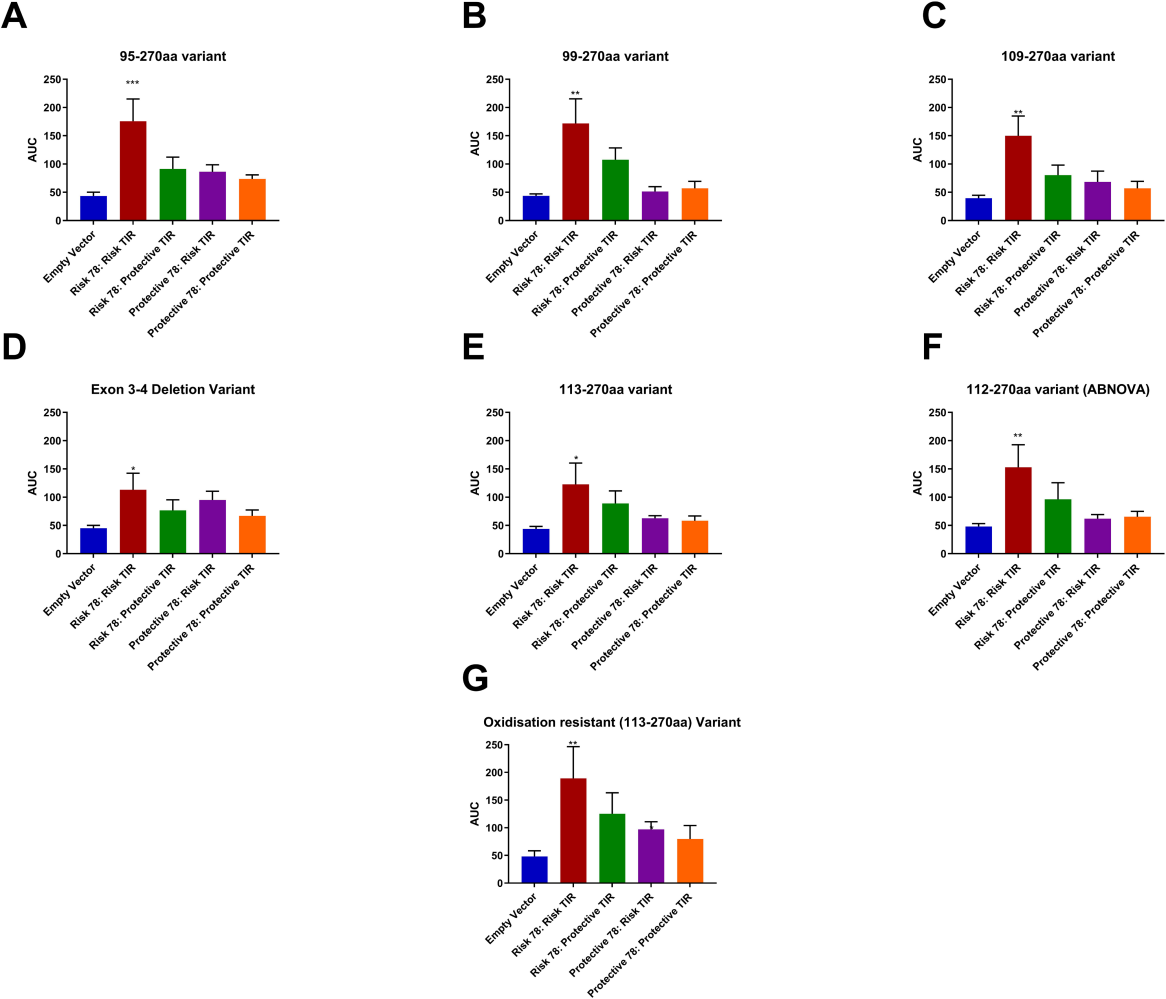
Area under the curve (AUC) analyses of SEAP data highlights the impact of both IL33 isoform and IL1RL1 haplotype on the magnitude of signalling. All IL33 isoforms are able to activate IL1RL1(Panels A–G) and, with the exception of IL33_OxR_, IL33‐driven activation of IL1RL1 requires the presence of the asthma risk alleles of both the extracellular IL1RL1‐variant Ala78Glu (rs1041973 [C]) and the TIR signalling domain haplotype (tagging SNP rs10192157 [C]). Maximal signalling can be observed for isoforms IL33_95–270_ (Panel A), IL33_99–270_ (Panels B) and IL33_106–270_ (Panels C) as well as IL33_OxR_ (Panel G). Data represent means ± SEM (*n* = 5 independent experiments). Comparisons were analysed using a Kruskal–Wallis test with Dunn's correction for multiple testing. *p < 0.05, **p < 0.01, ***p < 0.001.

**FIGURE 3:**
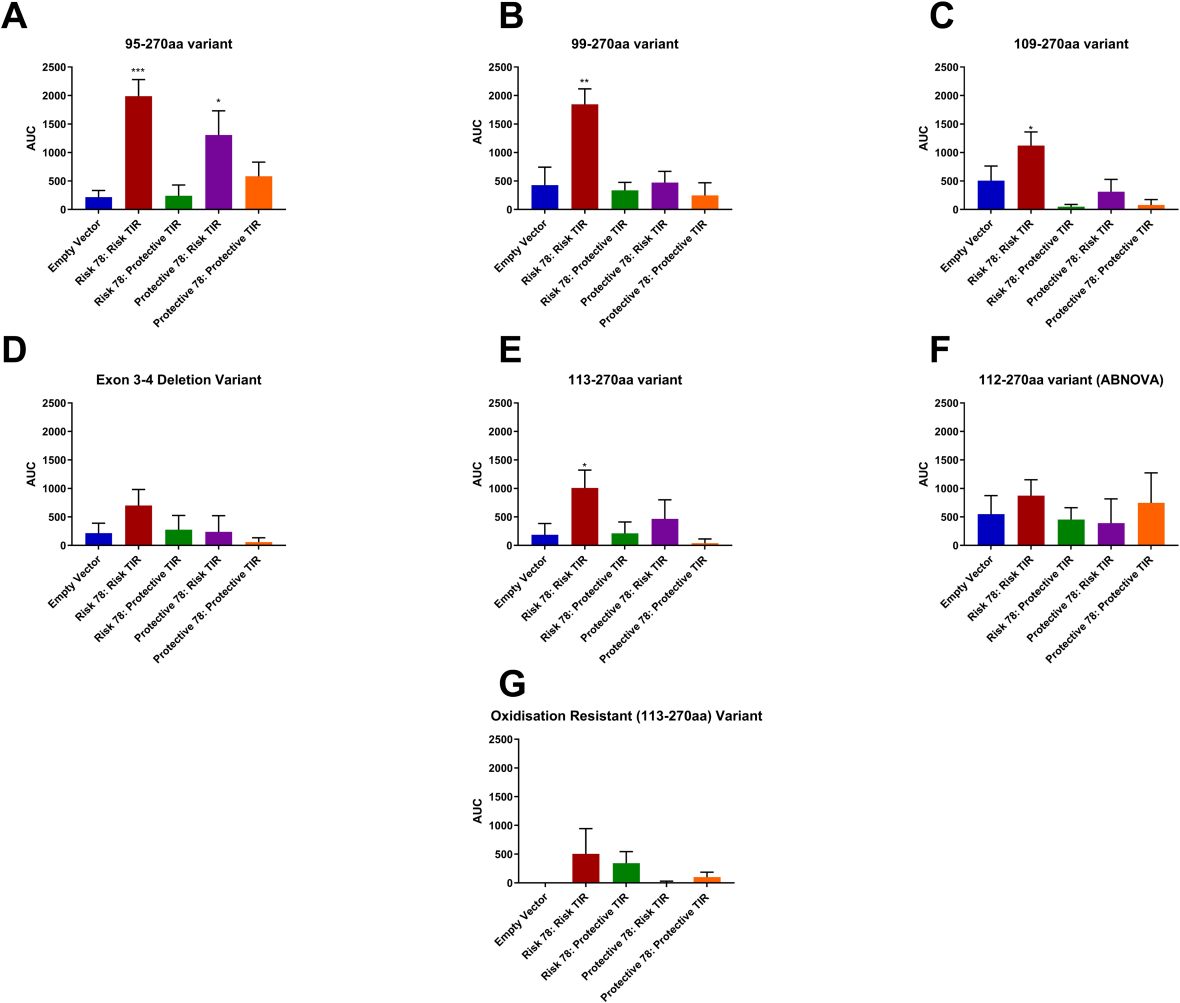
Area under the curve (AUC) analyses of IL8 data highlights the impact of both IL33 isoform and IL1RL1 haplotype on the magnitude of signalling. Only the longest isoforms of IL33 are able to elicit a secreted IL8 response IL33_95–270_ (Panel A), IL33_99–270_ (Panels B) followed by a moderate response from IL33_106–270_ (Panel C), IL33_113–270_ (Panels E). No response could be observed for IL33_Δ3,4_ (Panel D), IL33_112–270_ (Panel F) or IL33_OxR_ (Panel G). IL33 driven activation of IL1RL1 was shown to require the presence of the asthma risk alleles of both the extracellular IL1RL1‐variant Ala78Glu (rs1041973 [C]) and the TIR signalling domain haplotype (tagging SNP rs10192157 [C]). Data represent means ± SEM (*n* = 5 independent experiments). Comparisons were analysed using a Kruskal–Wallis test with Dunn's correction for multiple testing. *p < 0.05, **p < 0.01, ***p < 0.001.

To visualise these differences between carriers of the risk–risk haplotype and carriers of other combinations of the extracellular and TIR domain variants, we completed area under the curve analyses for SEAP (Figure 2) and IL8 production (Figure 3). As clearly shown in Figure 2, only carriers of the risk–risk haplotype demonstrate a significant response to IL33 isoforms based on the SEAP reporter system. This observation was universally observed, with the more potent isoforms (e.g., IL33_95–270_) showing a greater magnitude of difference between carriers and noncarriers of the risk–risk haplotype due to the greater response per se to this isoform (Figure 2A). As previously, we measured IL8 production, confirming that the risk–risk haplotype drives maximal signalling in cells carrying this receptor, using innate cell machinery, particularly for IL33_95–270aa_ and IL33_109–270aa_ (Figure 3).

### 3.3 Attenuated Signalling Is Observed in Carriers of a Single Asthma Risk Variant for Selected IL33 Isoforms

When measuring IL1RL1 signalling through secreted IL‐8 levels, we observe signalling with IL33_95–270_ (Figure 1 Panel B; 25 and 50 ng/mL [*p* < 0.0001]) and IL33_OXR_ (Figure 2 Panel N; *p* < 0.01) in the presence of the IL1RL1 protective allele for the extracellular variant (rs1041973 [A]) with the asthma risk TIR signalling domain haplotype (rs10192157 [C]).

When considering the SEAP activity assay as a measurement for NF‐κB signalling, IL33_99–270_ generated a response in the presence of the asthma risk allele (A) of rs1041973 with the asthma protective TIR signalling domain haplotype (rs10192157 [T]). Both the IL8 and SEAP identified responses only occurred at higher IL33 doses (25–50 ng/mL) and neither observation was replicated in the complimentary assay.

Area under the curve analyses were complementary to these analyses, highlighting multiple risk allele effects (Figures 2 and 3). For example, when using IL8 as a more physiologically relevant measure, there was measurable activity for the E78A protective:TIR domain risk haplotype, suggesting that the risk TIR domain is sufficient to generate a response in the presence of the extracellular protective variant, based on cytokine production for this potent IL33 isoform (Figure 3A).

### 3.4 Dose–Response Curves Highlight the Shift in EC_50_ for Carriers of the Risk–Risk Haplotype

To determine IL33 isoform sensitivity to receptor haplotypes, we calculated the EC_50_ value of each isoform when activating IL1RL1 (Table 1). EC_50_ values could only be determined for cell populations carrying the risk:risk (SEAP and IL8) and E78A risk:TIR domain protective (SEAP) haplotypes, with other responses being of insufficient magnitude to generate meaningful calculations. These data highlight muted responses for haplotypes containing protective alleles, with carriers of the risk: risk haplotypes generating higher EC_50_ values to a magnitude of 3 (IL33_95–270_) to 14 (IL33_113–270_) fold (Table 1). We identify that IL33_95–270_ generates the greatest activity in both SEAP and IL‐8 assays, SEAP EC_50_: 6.99 pM, *p* = 2.9 × 10^−3^, Panel B, IL8 EC_50_: 0.74 pM, *p* < 1.0 × 10^−4^.

We next investigated whether changes in the TIR signalling domain effected IL33 efficacy. SEAP EC_50_ values for each IL33 isoform, when stimulating IL1RL1 carrying the asthma risk allele for rs1041973 (C) and either the asthma risk or protective TIR signalling domain haplotype were compared, and no statistically significant difference was observed (Table 1). No EC_50_ values were calculated for models consisting of IL1RL1 carrying the protective allele (A) for the extracellular variant (rs1041973), due to a lack of a sufficient signal to plot meaningful EC_50_ curves.

### 3.5 Primary Bronchial Epithelial Cells From Asthma Patients Carrying the *IL1RL1* Variants Confirm Recombinant Cell Work

**FIGURE 4:**
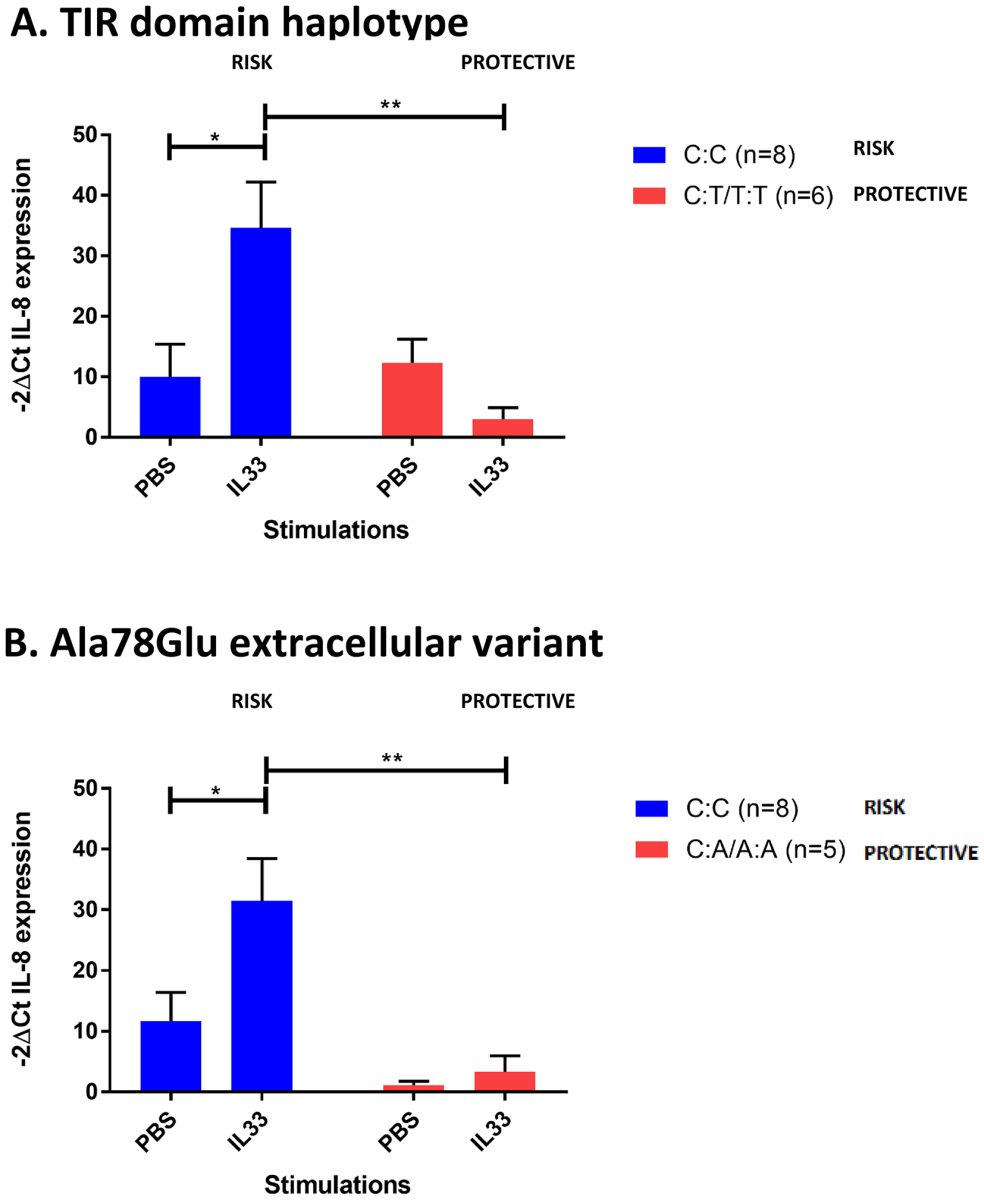
Primary bronchial epithelial cells isolated from patients with asthma that carry the protective extracellular or protective TIR domain variant show minimal response to IL33. Interleukin 8 (IL8) mRNA levels were elevated in bronchial epithelial cells taken from asthma patients that carry the asthma risk allele of the TIR signalling domain as tagged by the SNP rs10192157 (C Allele; Panel A) or the Ala78Glu extracellular variant (C Allele; Panel B) following a 24 h stimulation with 50 ng/mL IL33_112–270_, when compared to the vehicle control (PBS) (*p* < 0.05). *p < 0.05, **p < 0.01. Comparisons were analysed using a two‐way ANOVA.

Primary bronchial epithelial cells carrying either the TIR domain risk haplotype or the Glu78 extracellular risk amino acid had a greater response to IL33_112–270aa_, as determined by IL‐8 mRNA levels (Figure 4A,B).

### 3.6 Modelling the Structural Impact of Nonsynonymous Amino Acid Changes Identifies a Likely Disruption of IL33 Binding due to Changes in the Extracellular Domain

**FIGURE 5:**
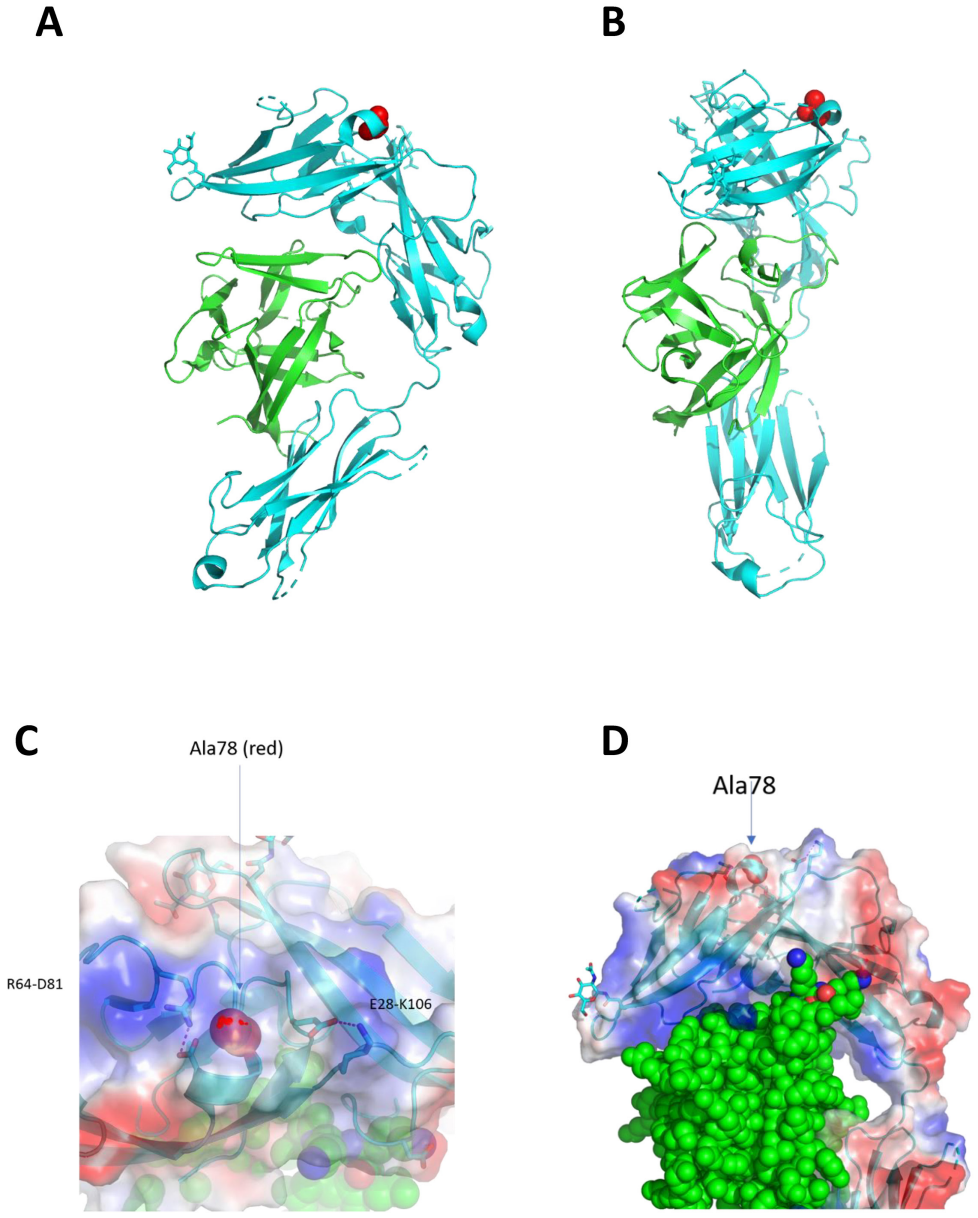
Topology diagram illustrating the structure of IL1RL1 in relation to the Ala78Glu variant illustrated as red spheres and present at the N‐terminal of the receptor, presented from two separate angles (Panels A,B). The Ala78 variant is shown as being partially buried by the turn of the short alpha helix of IL1RL1 (Panel C) and in relation to two salt bridges (R64‐D81 and E28‐K106). IL33 is shown in green. The substitution of Ala78 to Glu78 is likely to disrupt these salt bridges (depicted using red dotted lines) (Panel C) and through changes in receptor conformation can affect the ability of the IL33 ligand binding site to bind IL33 (Panel D). Disruption of the IL33 ligand binding site is likely to effect downstream IL1RL1 signalling, including NF‐κB signalling.

To provide a structural understanding of the loss or gain of function caused by Ala78 versus Glu78, we examined the context of the amino acid 78 residue in the available crystal structures of the IL1RL1 N‐terminal domain. The residue is located at the end of a short alpha helix and is present on the surface of IL1RL1 and is partially buried by surrounding amino acids. Although coding for a potentially surface‐exposed amino acid, this residue is in a crowded area between two salt bridges (Figure 5). Modelling the asthma risk (Glu) residue in place of Ala78 appears to alter the local structure and so may influence the electrostatics of the region pertaining to the Arg64‐Asp81 and Glu28‐Lys109 salt bridges and therefore indirectly modify ligand binding through overall disruption of the shape/conformation of the IL33 binding site. The TIR domain amino acid substitutions have been modelled in detail previously [10]. The Gln501Arg variant maps to the aD helix of the TIR domain, and the Ala433Thr‐variant maps to the aB helix close to the B–B loop. These regions have been implicated in a TIR domain dimerisation which may structurally underpin the changes in IL1RL1 signalling we and others have observed.

## 4 Discussion

We hypothesised that both IL33 isoforms present and IL1RL1 polymorphic variation determine the overall IL33/IL1RL1 pathway activity. We demonstrate that all IL33 isoforms have biological activity, activating NF‐κB signalling, with potential cooperative activation of the activator protein 1 (AP‐1) transcription factor due to known crosstalk between the signalling pathways, particularly in TNFR‐associated factor (TRAF) mediated signalling, such as in response to TNF and other proinflammatory cytokines [20]. IL33_95–270_ and IL33_109–270_ demonstrating maximal signalling, while IL33_Δ3,4_ and IL33_112–270_, had modest effects. Significantly, our data identified that maximal IL1RL1 signalling occurs in cells carrying both asthma *IL1RL1* risk variants in the extracellular and TIR domains, that is, the protein sequence that contains Ala78 and Ala433‐Glu501‐Thr549‐Leu551 together. These data indicate that IL1RL1 signalling intensity, and by inference downstream Type 2 inflammation, is driven by IL33 isoform type present and by *IL1RL1* asthma risk associated nonsynonymous variants. We suggest that this novel finding may be particularly important in asthma patients that carry *IL1RL1* asthma risk alleles and have a lung microenvironment that promotes elevated levels of cleaved IL33 isoforms, leading to a more IL33‐driven disease amenable to targeting.

### 4.1 IL33 Isoforms Show Differential Ability to Activate IL1RL1

The strongest signalling Isoforms IL33_95–270_ and IL33_109–270_, are generated through inflammatory proteases such as mast cell chymase [16], neutrophil elastase and cathepsin‐G [13] as well as allergen proteases from fungi, HDM and subtilisin [15]. This supports reports of elevated IL33 in the epithelial and subepithelial compartments of epithelial biopsies, collected from mild allergic asthmatics following allergen challenge [21]. IL33_95–270_ and IL33_109–270_ activate ILC2 and MC/9 mast cells [16], suggesting that a greater abundance of these isoforms may be present in allergic asthma driven by fungal and HDM triggers as well as in Type 2 and neutrophilic asthma. By inference, an increased role for the IL33/IL1RL1 signalling pathway may be present due to the increased abundance and therefore signalling response to these isoforms in some but not all asthma patients. Importantly, our data are in excellent agreement with IL33 isoforms generated by mast cell proteases (IL‐33_95–270_, IL‐33_107–270_ and IL‐33_109–270_) having up to 30‐fold increased potency to activate ILC2s [16].

Conversely, we identify limited responses to IL33_Δ3,4_ and IL33_112–270_, the latter having been widely used in the literature. Both IL33_112–270_ isoforms, one manufactured by us and one commercially available, gave measurable but modest responses in our IL1RL1 recombinant model. IL33_Δ3,4_ elicits a limited, albeit not statistically significant response in contrast to earlier reports [17, 22]. However, it is important to note that while our data suggest a greatly reduced/background activity for this isoform we cannot exclude differences in the preparation of this recombinant protein and/or the sensitivity of the different cell/assay models used.

Investigation of IL33_OxR_, an IL33_112–270_ variant modified to overcome loss of activity through oxidation, identified that while an NF‐κB activation response in excess of that reported for IL33_95–270_ at higher doses (≥10 ng/mL), the IL8 response curve reflecting IL33 activity shifted to the left and lost response at higher concentrations. IL33_OxR_ may therefore be eliciting a nonphysiological signalling response.

### 4.2 IL1RL1 Nonsynonymous Variation Associated With Asthma Risk Modulate Cell Responses to IL‐33

Our results show that maximal IL1RL1 signalling requires the presence of both TIR signalling domain and rs1041973 asthma risk variants, which is a novel finding. These distinct nonsynonymous *IL1RL1* variants appear to act in synergy for maximal IL33 activation of its receptor, where the absence of either attenuates signalling. Of these, the risk TIR domain haplotype appears to be of greater importance to IL1RL1 signalling, as modest signalling activity occurs even in the presence of the protective rs1041973 allele (A), while the absence of the TIR signalling domain risk haplotype resulted in a complete loss of signal, regardless of the rs1041973 allele carried.

These data are in agreement with previous studies, including our own, that showed the presence of the asthma risk haplotype in the TIR domain (Ala433‐Glu501‐Thr549‐Leu551) drives augmented IL1RL1 signalling [10, 11, 12]. The findings for the Ala78Glu variant are novel and add a significant advance in our understanding of how IL1RL1 signalling may be modulated by naturally occurring nonsynonymous variation.

### 4.3 Modelling Identifies That Changes Driven by the Ala78Glu Extracellular Variant May Inhibit IL33 Binding

Through modelling of the available IL1RL1 crystalline structure we identified that changing the Ala78 residue to the Glu78 variant resulted in an alteration of the main alpha chain structure which is likely to disrupt the electrostatics of two salt bridges observed in the region (Arg64‐Asp81 and Glu28‐Lys109). This change to salt bridge structure could have knock‐on effects on IL1RL1 crystalline structure conformation and affect the IL33 binding site to an extent that ligand binding may be disrupted. These changes to IL33 ligand binding capabilities may explain the observed loss of signalling in IL1RL1 containing the Ala residue. Similarly, loss of signalling in carriers of the protective TIR domain haplotype may be due to loss of efficient domain dimerisation required for optimal signalling, particularly around the Gln501Arg and Ala433Thr residues, hence the greater magnitude of the risk haplotype versus the extracellular variation.

### 4.4 Identifying an IL33 Driven Asthma and Target Population for Anti‐IL1RL1 or Anti‐IL33 Therapy

These cell‐based data suggest that the airway microenvironment, which drives the formation of different IL33 isoforms, in combination with *IL1RL1* nonsynonymous genetic variation, may determine the inflammatory response in specific asthma patients. This may therefore identify patients with more IL33‐relevant asthma. With this in mind, it is important to note that there are differences in the frequency of the risk–risk haplotype in populations with different ancestry, for example, EUR: 50.8%, AMR:65%, EAS:81.7% and SAS:73.6% populations and in particular lower frequencies in African populations of 17.5%. Therefore, we anticipate that while the majority of worldwide populations will be susceptible to IL33‐dependent IL1RL1 activation due to carrying the risk: risk haplotype, and therefore likely be amenable to anti‐IL1RL1 and/or anti‐IL33 therapy, we expect people of African ancestry may be naturally protected from the activation of inflammation by IL33.

In conclusion, we have identified that IL33 isoforms and nonsynonymous genetic variation present in the *IL1RL1* are critical for determining the IL1RL1 signalling response and by inference the magnitude of the anticipated Type 2 inflammation. The contrast between carriers of the risk: risk *IL1RL1* haplotype versus those carrying either of the protective alleles was striking. These new findings may facilitate the identification of patients more (or less) amenable to IL33/IL1RL1 blocking strategies targeting this pathway for clinical benefit in asthma.

## Author Contributions

I.S., M.A.P., G.H.K. and M.C.N. conceived and planned the experiments. M.A.P. and J.E. carried out the experiments, data analysis. I.S., M.A.P., G.H.K., M.C.N., J.E. and K.A. contributed to interpretation. M.E.K., D.S., C.B., K.A. and A.J.V.O. provided materials for the study. M.A.P. wrote the manuscript with support from I.S., G.H.K. and M.C.N. I.S., G.H.K., M.C.N. and A.J.V.O. conceived the original idea. I.S., I.H., G.H.K., M.C.N., A.J.V.O. and K.A. supervised the project. All authors provided critical feedback and helped shape the research, analysis and manuscript.

## Conflicts of Interest

M.A.P., I.H., I.S., G.H.K. and M.C.N. report funding from GlaxoSmithKline related to this manuscript. M.E.K. reports funding from GlaxoSmithKline and the European Respiratory Society related to this manuscript. I.H. and I.S. declare further research collaborations with GSK and Boehringer Ingelheim. M.C.N. reports support (unrestricted research grants) from GlaxoSmithKline plc and the Ministry of Economic Affairs and Climate Policy of the Netherlands (PPP allowance). A.J.V.O. declares himself as a shareholder of GSK stock. G.H.K. declares membership to advisory boards to GSK and Pure‐IMs, funding pertinent to this manuscript from the Lung Foundation of the Netherlands and GSK and funding external to this work from the TETRI foundation, Vertex, TEVA the Netherlands, European Union (H2020) and the Ubbo Emmius Foundation. K.A., S.B., E.C. and M.E. are employees of GSK but declare no further conflict of interest relating to this publication. J.E., D.S. and C.B. declare no conflicts of interest.

## Supporting information


Appendix S1.

## Data Availability

The data that support the findings of this study are available from the corresponding author upon reasonable request.
