# Supplementary material for: Epithelial Interleukin‐1 Receptor‐Like‐1 Activation Is Contingent on Interleukin‐33 Isoforms and Asthma‐Related Receptor Variation
Source: Clin Exp Allergy. 2024 Sep 20;54(12):984–95. doi: 10.1111/cea.14562 (PMC11629065; doi:10.1111/cea.14562)
Supplement: Supplementary file 1 — Appendix S1. [file CEA-54-984-s001.docx]

**Supplemental Methods**

## Primary bronchial epithelial cell isolation and culture

Moderate-severe asthma patients were recruited from Nottingham University Hospitals and Glenfield Hospital, Leicester, UK as per treatment step outlined in the Global Initiative for Asthma (GINA) guidelines. These studies were conducted in accordance with the amended Declaration of Helsinki, ethics reference 11/EM/0062 and 12/EM/0059 (Nottingham) and ethics reference 4977/project approval number 6347 (Leicester). Written informed consent was obtained from all patients. Human bronchial epithelial cells were obtained from bronchial brushings as previously described(30). Passage 3 cells were cultured on PureCol Type-I Bovine collagen (Advanced BioMatrix, 5005-B) in fresh medium (Pneumacult-Ex; StemCell, 05008) supplemented with Hydrocortisone Stock Solution (StemCell; 07925) and antibiotic-Antimycotic solution (ThermoFisher Scientific; 15240062). Cells were allowed to reach 90% confluence in submerged culture and incubated with 50ng/ml human recombinant IL33_112-270aa_ (ABNOVA; P3638) or sterile PBS for 24 hours. RNA lysates were collected as previously described(31)

## Preparation and transfection of plasmids

*IL1RL1* overexpression was carried out using the pCDH-CuO-MCS-IRES-RFP SparQ™ Cloning and Expression Lentivector (SBI Biosciences; QM531A-2). The open reading frame of *IL1RL1* transcript variant 1 (NM_016232.5) was amplified commercially using the pRP[Exp]-EGFP-CMV vector (VectorBuilder). Four different open reading frames were synthesised to contain either allele of variant of rs1041973 (Risk = Ala, Protective = Glu) and either haplotype of the TIR signalling domain (Risk = Ala433-Glu501-Thr549-Leu551 or Protective = Thr433-Arg501-Ile549-Ser551 haplotypes), i.e. risk:risk, protective:risk, risk:protective, protective:protective. Each ORF was cloned using the NheI and BamHI into pCDH-CuO-MCS-IRES-RFP vector using restriction enzymes and T4 ligase (Promega). All plasmids were sequence verified.

## Genotype and haplotype frequencies in populations of different ancestry

In order to evaluate the impact of the functional effects observed in vitro in populations of different ancestry that may have different frequencies of these variants we determined both the genotype and haplotype frequency of the extracellular domain, rs1041973 (Ala78Glu) and the TIR series of amino acid variants ((rs4988956 (Ala433Thr) , rs4988957 (Asn455Asn), rs10192036 (Gln501Lys), rs10204137 (Gln501Arg), rs4988958 (Ser525Ser), rs10192157 (Thr549Ile), rs10206753 (Leu551Ser)). The asthma risk haplotype rs1041973C and rs10192157C frequency was compared across different ancestry using data from the LDpair tool (<https://ldlink.nih.gov/?tab=home>, accessed 5^th^ February 2024).

## Statistical analysis

Statistical analysis, EC_50_ calculations and area under the curve calculations were carried out using the PRISM software package (GraphPad Software, Inc. Version 7.03). Longitudinal data was analysed using a repeated measures Two-way ANOVA with post-hoc Sidak correction for multiple testing. Single time-point testing was carried out using Kruskal-Wallis or Mann-Whitney tests as relevant to the number of compared data groups. A P<0.05 was considered significant.

**Supplemental Results**

## Genotype and haplotype frequency differences based on ancestry may highlight populations with heightened IL33 responsiveness.

The genotype frequency of the extracellular risk allele, rs1041973C (Glu) was 0.76 in Europeans, 0.43 in Africans, 0.86 in South Asians, 0.85 in East Asians and 0.80 in Ad Mixed Americans. For rs10192157C that tags the TIR domain risk haplotype; 0.60 in Europeans, 0.25 in Africans, 0.79 in South Asians, 0.88 in East Asians and 0.71 in Ad Mixed Americans. The frequency of the risk:risk haplotype was 0.51 in Europeans, 0.18 in Africans, 0.74 in South Asians, 0.74 in East Asians and 0.65 in Ad Mixed Americans. These data suggest that the asthma risk alleles are the common alleles found in the European population suggesting functionality is more commonly retained.

**Supplemental Figures**


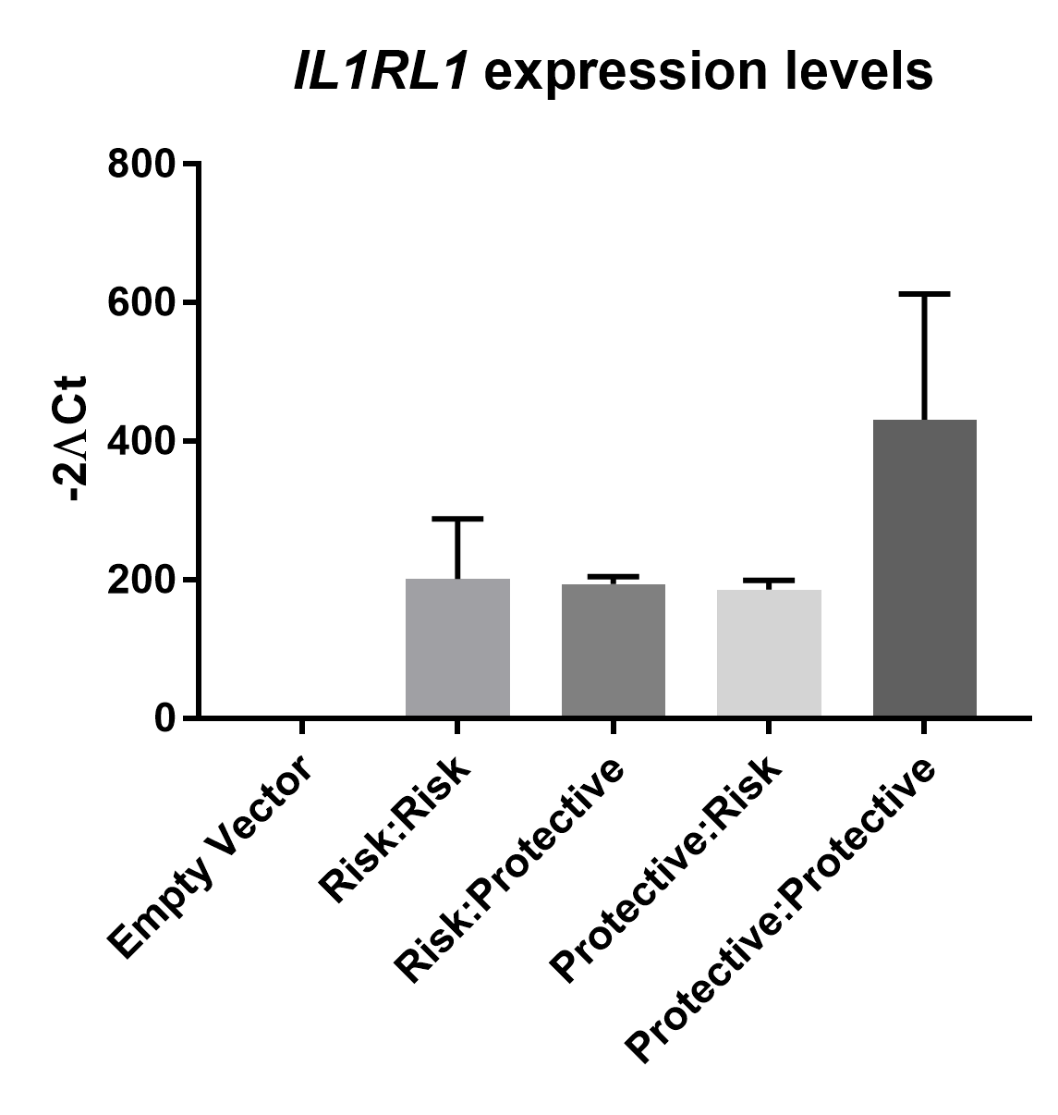


Supplemental Figure 1: Comparison of IL1RL1 mRNA expression levels following transient transfection of IL1RL1 overexpression constructs. No statistically significant difference in IL1RL1 was observed across all four constructs (P>0.05), confirming that observed changes in IL1RL1 signalling patterns is unlikely to be driven by differential receptor expression.


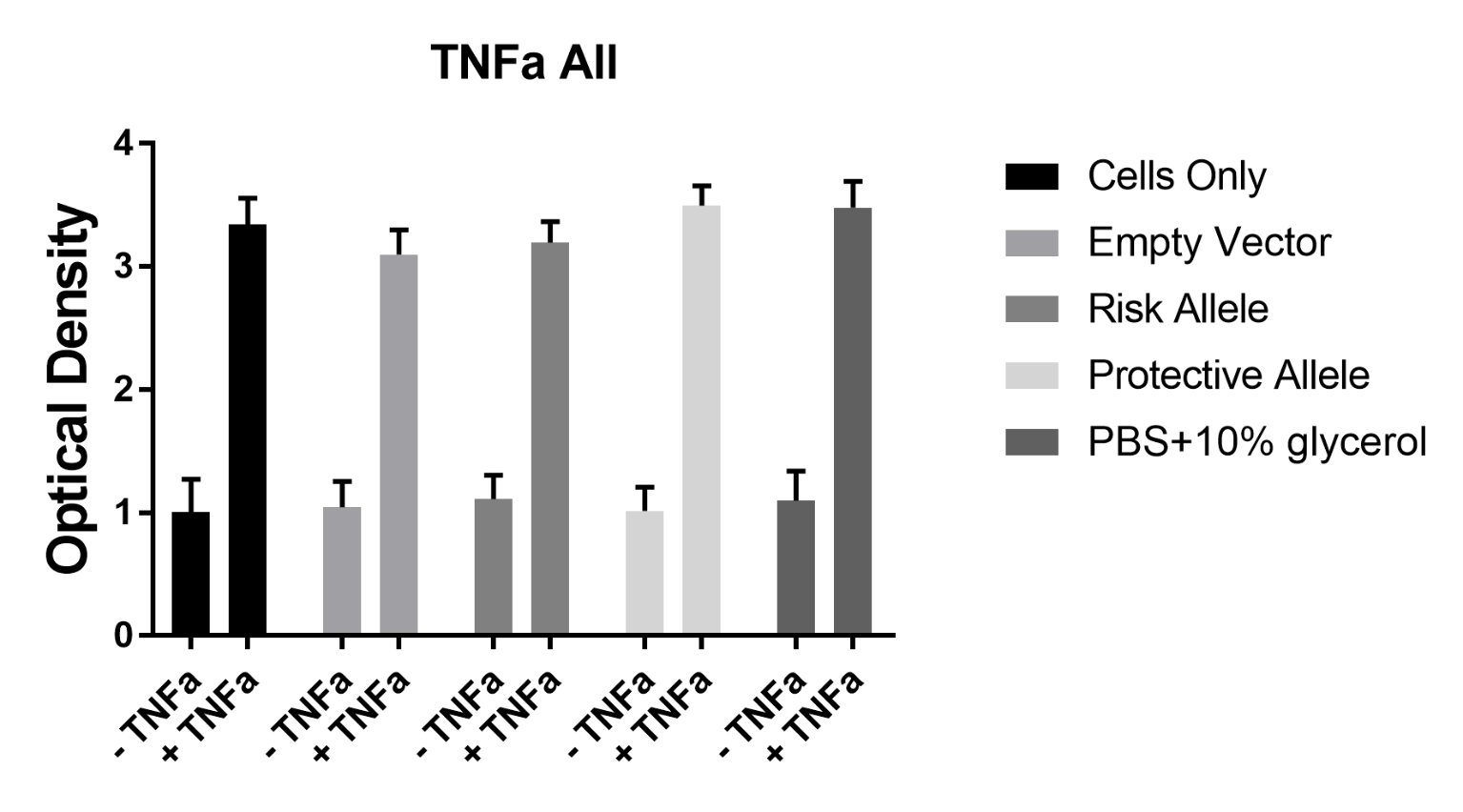
Supplemental Figure 2: Comparison of TNFα activity across different IL1RL1 expression cell lines identifies that while a significant change in SEAP expression occurs following stimulation with 1ng/ml of TNFα in all cell lines (P=0.0008), no discernible difference in the degree of response to TNFα is observed across the different cells containing different IL1RL1 vectors (P=0.49). Comparisons were analysed using a 2-way ANOVA with Sidak correction for multiple testing.

**A B**


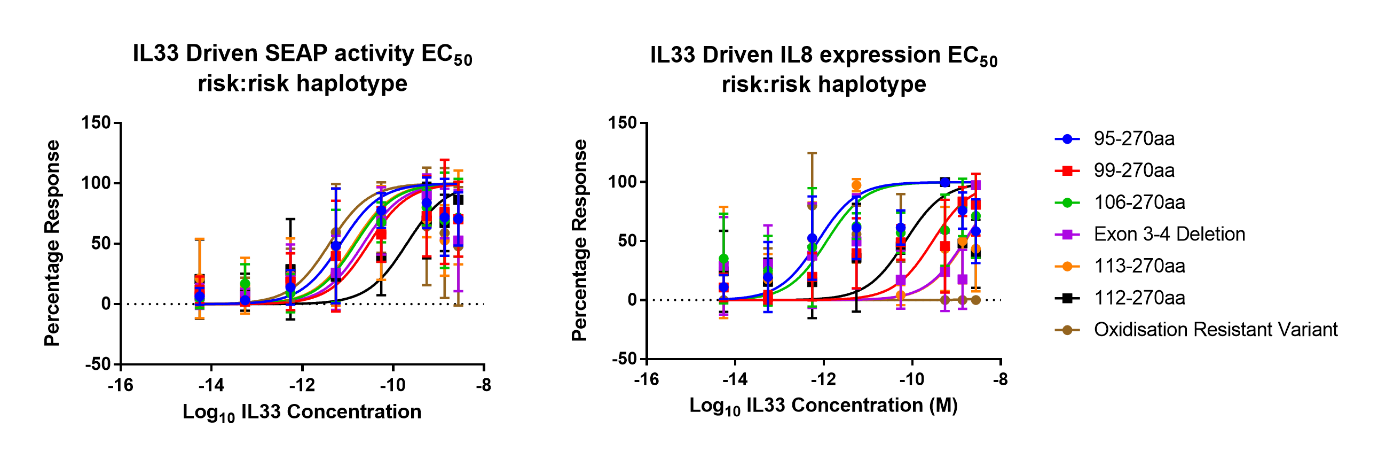


Supplemental Figure 3: Different IL33 isoforms have a different EC_50_ value. HEK293 cells carrying the asthma risk alleles for the extracellular 78 variant Ala78Glu (rs1041973 (C)) and the TIR signalling domain haplotype (tagging SNP rs10192157 (C)). Graphs represent SEAP (Panel A) and IL8 ELISA (Panel B) outputs as determinants of IL1RL1 signalling activity. In both cases a statistically significant variation in EC50 values could be observed across different IL33 isoforms (SEAP: P=0.027, IL8: P<0.0001). Increased sensitivity was observed for IL33_95-270_ and IL33_106-270_ compared to all other isoforms across both assays. Although presenting with strong activity in SEAP measurements (Panel A), an EC50 curve could not be calculated for IL33_OxR_ when considering IL8 responses (Panel B), suggesting a disconnect between the SEAP and IL8 assays for this IL33 isoform.
